# Preclinical Evidence of Paeoniflorin Effectiveness for the Management of Cerebral Ischemia/Reperfusion Injury: A Systematic Review and Meta-Analysis

**DOI:** 10.3389/fphar.2022.827770

**Published:** 2022-04-08

**Authors:** Anzhu Wang, Wei Zhao, Kaituo Yan, Pingping Huang, Hongwei Zhang, Xiaochang Ma

**Affiliations:** ^1^ Xiyuan Hospital, China Academy of Chinese Medical Sciences, Beijing, China; ^2^ Graduate School, China Academy of Chinese Medical Sciences, Beijing, China; ^3^ Yidu Central Hospital of Weifang, Weifang, China; ^4^ National Clinical Research Center for Chinese Medicine Cardiology, Beijing, China

**Keywords:** preclinical evidence, potential mechanisms, paeoniflorin, cerebral ischemia-reperfusion injury, animal studies

## Abstract

**Background:** Vessel recanalization is the main treatment for ischemic stroke; however, not all patients benefit from it. This lack of treatment benefit is related to the accompanying ischemia-reperfusion (I/R) injury. Therefore, neuroprotective therapy for I/R Injury needs to be further studied. *Paeonia lactiflora Pall.* is a commonly used for ischemic stroke management in traditional Chinese medicine; its main active ingredient is paeoniflorin (PF). We aimed to determine the PF’s effects and the underlying mechanisms in instances of cerebral I/R injury.

**Methods:** We searched seven databases from their inception to July 2021.SYRCLE’s risk of bias tool was used to assess methodological quality. Review Manager 5.3 and STATA 12.0 software were used for meta-analysis.

**Results:** Thirteen studies, including 282 animals overall, were selected. The meta-analyses showed compared to control treatment, PF significantly reduced neurological severity scores, cerebral infarction size, and brain water content (*p* = 0.000). In the PF treatment groups, the apoptosis cells and levels of inflammatory factors (IL-1β) decreased compared to those in the control groups (*p* = 0.000).

**Conclusion:** Our results suggest that PF is a promising therapeutic for cerebral I/R injury management. However, to evaluate the effects and safety of PF in a more accurate manner, additional preclinical studies are necessary.

## Introduction

Stroke is the second leading cause of death worldwide, and 84.4% of stroke cases are related to ischemia (Collaborators, 2019). Although mechanical thrombectomy and intravenous thrombolysis have been widely recommended and used in the treatment of acute ischemic stroke patients, the treatments are not effective in all patients (Powers et al., 2018; Liu et al., 2020). Besides some known complications, subsequent ischemia/reperfusion (I/R) injury may be the most important factor resulting in a poor prognosis (Kalogeris et al., 2016). Cerebral I/R injury is characterized by a biochemical cascade of ischemic reactions that result in brain tissue deterioration, limiting the beneficial effects of vascular recanalization (Hu et al., 2015). I/R injury is involved in some complicated pathophysiological mechanisms, such as the release of excitatory neurotransmitters, the acceleration of Ca^2+^ influx into cells, free radical damage, neuronal apoptosis, neuroinflammation, and fat decomposition (Siesjo, 1992; Wang and Lo, 2003; Wu et al., 2018). Therefore, currently used neuroprotective therapies aimed at I/R injury management need further research.

Animal models of ischemic stroke are crucial for determining the pathophysiology of ischemic stroke and creating novel stroke therapies. *In vivo* stroke models are now predominantly mice and rats, which is understandable given the lower costs of procurement and maintenance, as well as the ease of monitoring and tissue processing (Sommer, 2017). The intraluminal suture middle cerebral artery occlusion (MCAO) model, which does not need craniectomy, is the most commonly used experimental model for ischemic stroke in rats (Alrafiah, 2021).

**FIGURE 1:**
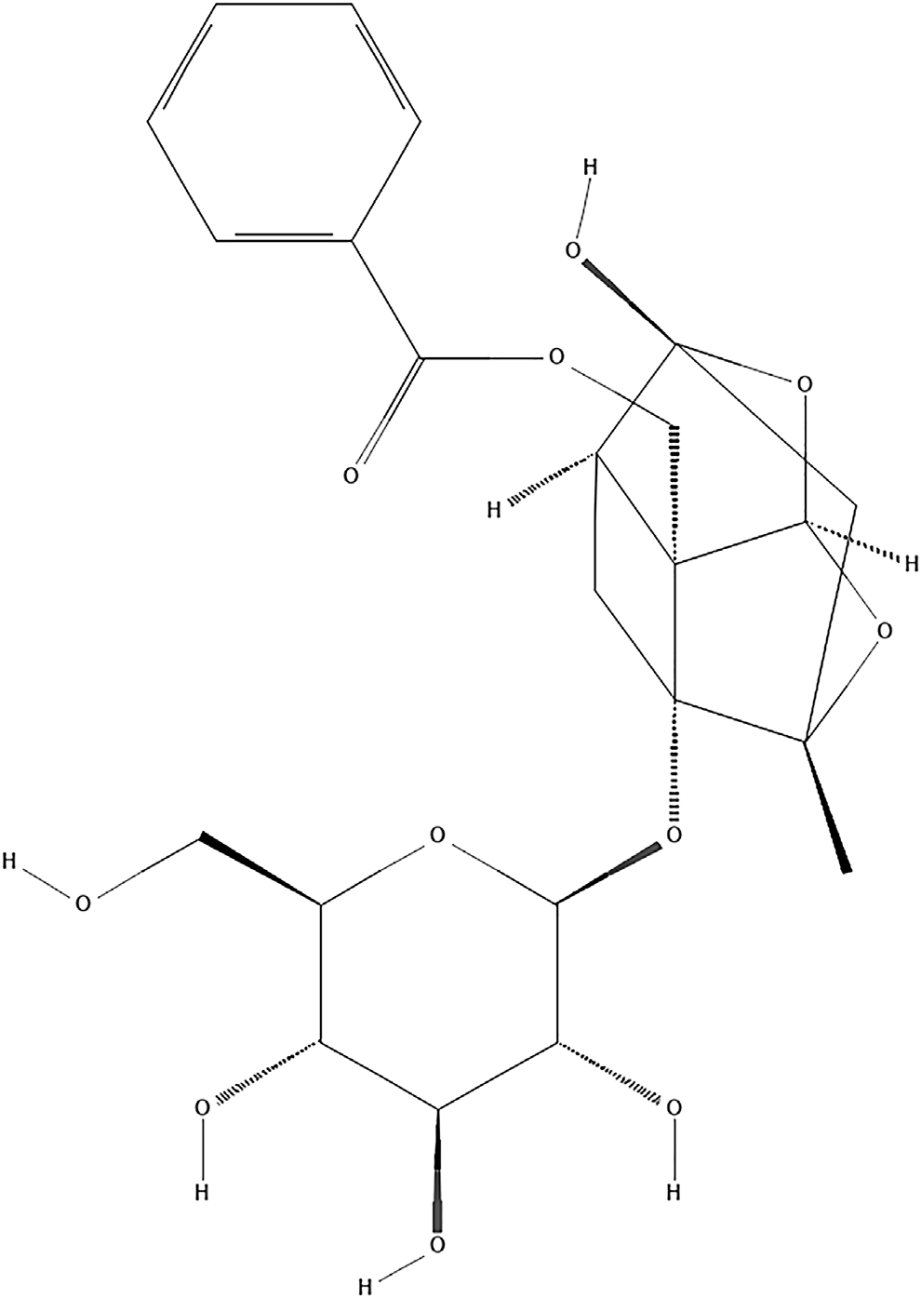
Chemical structure of paeoniflorin.

Paeoniflorin (PF, C23H28O11; Figure 1) is a natural compound derived from *Paeonia lactiflora Pall.* (Family Ranunculaceae, molecular mass: 480.5) (Sterne et al., 2019). Traditional Chinese medicine (TCM) theory believes that *Paeonia lactiflora Pall.* has the function of clearing heat and cooling blood, promoting blood circulation and removing blood stasis. As an important component of traditional TCM compounds such as Buyanghuanwu Decoction and Huangqi Guizhi Wuwu Decoction, *Paeonia lactiflora Pall.* is widely used in stroke treatment in China (Chen et al., 2019; He et al., 2021). The neuroprotective benefits of PF have received a lot of attention in recent years. At present, the effects and mechanisms of PF on the central nervous system mainly come from *in vitro* experiments on nerve cells (such as primary cortical and hippocampal neurons, PC12 cells, and microglia cells) and *in vivo* investigations (Hu et al., 2018; Cong et al., 2019; Cheng et al., 2021). Studies have confirmed that PF can cross the blood-brain barrier, and its mechanism may be related to the mode of cell death, inflammation, oxidative stress and epigenetics (Jiao et al., 2021). Furthermore, PF has demonstrated its potential therapeutic utility in preventing I/R damage in a variety of tissues (Xie et al., 2018; Wen et al., 2019).Because of the complexity of clinical medicine, many differences between preclinical and clinical studies have prevented the further application of PF. A systematic review can not only offer reliable evidence but also facilitate the choice of an appropriate medicine for clinical experiments (van Luijk et al., 2013). However, no thorough examination of the effectiveness of PF pooled in preclinical investigations has been done to date. For this reason, we conducted a full systematic review and meta-analysis to evaluate PF’s effects in small-animal research on brain I/R Injury.

## Materials and Methods

This is a systematic review and meta-analysis based on Preferred Reporting Items for Systematic Reviews and Meta-Analyses (PRISMA).

### Search Strategy

We systematically searched the following seven databases: China National Knowledge Infrastructure, Wanfang Database, VIP Database, PubMed, Cochrane Library, Web of Science, and EMBASE from their inception to July 2021. The search terms used were as follows: (“Paeoniflorin” OR “Peoniflorin”) AND (“Brain Ischemia” OR “Ischemic Encephalopathy” OR “Cerebral Ischemia”) AND (“Reperfusion Injury” OR “Reperfusion Damage” OR “Ischemia-Reperfusion Injury”). In addition, all review articles, meeting abstracts, and their references were examined thoroughly without language limitations. The search target was research on animals.

### Inclusion and Exclusion Criteria

The inclusion criteria were as follows: (1) establishment of I/R experimental models through MCAO; (2) PF as the only consistent therapeutic medication and the use of placebo or no treatment in animals in the control group; and (3) animal research. The exclusion criteria were as follows: (1) establishment of I/R experimental models through other means; (2) PF not being the only intervention; (3) treatment of animals by using PF analogs; (4) literature with repetitive content; and (5) *in vitro* studies.

### Data Extraction

Two reviewers (Anzhu Wang and Pingping Huang) independently selected literature per the abovementioned criteria and resolved differences by discussion with assistance from a third reviewer (Xiaochang Ma). The following information was extracted from each study and is summarized in a table: (1) study features (names of the first authors and publication data); (2) animal characteristics including species, sex, weight, and age; (3) key elements of the MCAO model—the types of anesthetics used and duration of ischemia; (4) information about interventions—administration route, dose, and treatment time; and (5) mean and standard deviation values of the results. When findings were recorded at different time points, only those corresponding to the latest time point were considered. When different doses of a medicine were administered, the reviewers only record the highest dose. If data was reported in the form of a figure, the reviewers used a digital ruler, specifically the Adobe ruler, to determine the numerical values. If there were several publications with similar data, we only chose the earliest one or the one with the most samples.

### Quality Assessment

The included studies were assessed for bias by using the SYRCLE’s risk of bias tool by two independent reviewers (Anzhu Wang and Pingping Huang) (Hooijmans et al., 2014): which are: selection bias (sequence generation, baseline characteristics, and allocation concealment), performance bias (random housing and blinding of investigators), detection bias (random outcome assessment and blinding of the assessor to outcomes), withdrawal bias (availability of incomplete outcome data), selective reporting bias (selective outcome reporting), and other bias (other sources of bias). When one required standard was reached, one point was assigned. After evaluating 10 standards, each piece of literature was assigned a comprehensive quality score. Two reviewers, Anzhu Wang and Pingping Huang, resolved differences through discussion and with assistance from Xiaochang Ma, the third reviewer.

### Statistical Analysis

Reviewers adopted Review Manager 5.3 and STATA 12.0 for data analysis. Outcomes were presented as standardized mean differences with a 95% confidence interval. *p* < 0.05 indicated statistically significant. There was statistical heterogeneity between the Q test and I^2^ results for the literature assessed. *p* < 0.1 and I^2^ >50% were regarded to indicate significant heterogeneity; outcomes were assessed using a random-effects model. *p* > 0.1 and I^2^ ≤ 50% were regarded as indicating no heterogeneity; the outcomes were evaluated using a fixed-effects model. Potential publication bias was examined and evaluated by applying Egger’s test. Sensitivity and subgroup analyses for a single study were performed using Metaninf.

## Results

### Study Selection

**FIGURE 2:**
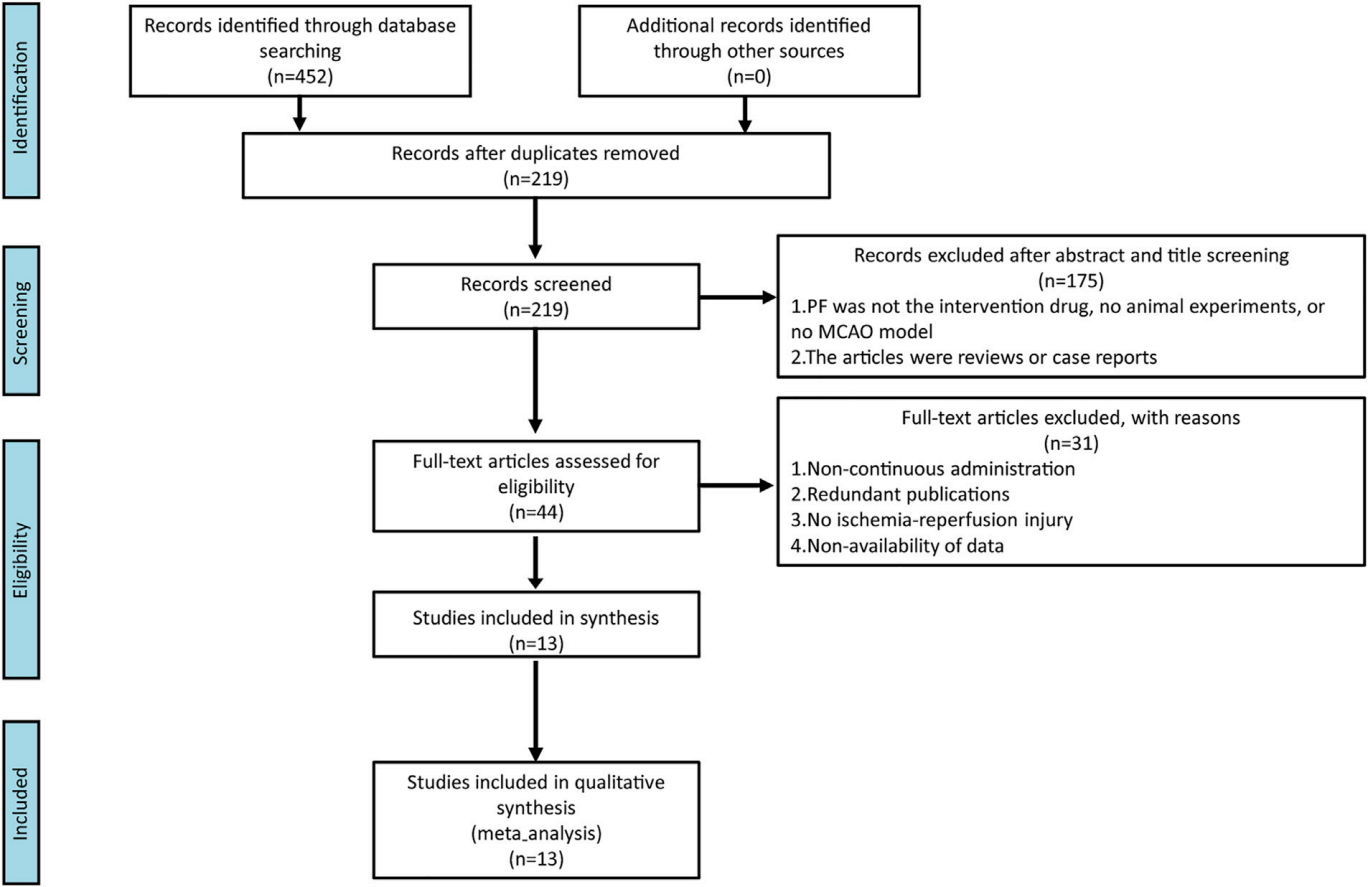
Flow diagram of the study-search process.

We identified 452 studies in the database search, and 219 studies remained after eliminating repeated studies. After reading titles and abstracts, 44 studies are considered. Overall, 175 pieces of literature were eliminated for the following reasons: (1) PF was not the intervention drug, no animal experiment, or no MCAO model; and (2) the articles were reviews or case reports. Finally, 13 were considered after reading the full text and 31 were eliminated. The reasons for exclusion were: (1) non-continuous administration; (2) redundant publications; (3) no ischemia-reperfusion injury; and (4) non-availability of data. The process of literature selection is shown in Figure 2.

### Study Characteristics

A total of 13 studies (Xiao, 2005; Wang, 2008; Tang et al., 2010; He, 2014; Mao et al., 2014; Rao, 2014; Zhang et al., 2015; Liu, 2016; Chu et al., 2017; Ko et al., 2018; Liao, 2018; Yu et al., 2018; Tang et al., 2021), including five English studies (Tang et al., 2010; Zhang et al., 2015; Chu et al., 2017; Ko et al., 2018; Tang et al., 2021) and eight Chinese studies (Xiao, 2005; Wang, 2008; He, 2014; Mao et al., 2014; Rao, 2014; Liu, 2016; Liao, 2018; Yu et al., 2018),were considered. The studies were published from 2005 to 2021. Five of these were master’s or doctoral theses (Xiao, 2005; Wang, 2008; He, 2014; Rao, 2014; Liao, 2018). All of them were concerned with 282 male Sprague–Dawley rats, whose weight varied from 180 to 350 g. In one study, pentobarbital sodium was used to anesthetize animals (Yu et al., 2018), while isoflurane was used in another (Ko et al., 2018). Chloral hydrate was used in the remaining 11 (Xiao, 2005; Wang, 2008; Tang et al., 2010; He, 2014; Mao et al., 2014; Rao, 2014; Zhang et al., 2015; Liu, 2016; Chu et al., 2017; Liao, 2018; Tang et al., 2021). Moreover, in five studies, PF was used before treatment (Xiao, 2005; Wang, 2008; He, 2014; Mao et al., 2014; Liu, 2016), and in seven, it was used after treatment (Rao, 2014; Zhang et al., 2015; Chu et al., 2017; Ko et al., 2018; Liao, 2018; Yu et al., 2018; Tang et al., 2021). In one study, it was used both before and after treatment (Tang et al., 2010). Furthermore, in nine out of 13 studies, intraperitoneal administration was adopted (Xiao, 2005; Wang, 2008; He, 2014; Zhang et al., 2015; Liu, 2016; Chu et al., 2017; Ko et al., 2018; Yu et al., 2018; Tang et al., 2021), while in three, intravenous tail injection was performed (Tang et al., 2010; Liao, 2018; Yu et al., 2018). In the final study, intragastric administration was adopted (Mao et al., 2014). Neurological severity scores (NSS) were reported in all studies. Three studies (Xiao, 2005; Tang et al., 2010; Mao et al., 2014) referred to the scoring method of Bederson et al. (1986), while three others (Chu et al., 2017; Ko et al., 2018; Tang et al., 2021) referred to the scoring method of Chen et al. (2001). In seven studies (Wang, 2008; He, 2014; Rao, 2014; Zhang et al., 2015; Liu, 2016; Liao, 2018; Yu et al., 2018), the scoring method of Longa et al. (1989) was used. Cerebral infarction size (CIS) was reported in nine studies (Xiao, 2005; Wang, 2008; Tang et al., 2010; He, 2014; Rao, 2014; Zhang et al., 2015; Liu, 2016; Liao, 2018; Yu et al., 2018). One out of the nine studies did not mention the exact method (Liu, 2016). In three of the nine studies, infarct area/contralateral brain area was used for CIS determination (Xiao, 2005; He, 2014; Rao, 2014). In the remaining five studies, CIS determination was based on infarction area/total brain area (Wang, 2008; Tang et al., 2010; Zhang et al., 2015; Liao, 2018; Yu et al., 2018). In five studies, the expression of the associated protein was determined by western blot (WB) (Xiao, 2005; Wang, 2008; He, 2014; Chu et al., 2017; Tang et al., 2021), while in two, reverse transcription-polymerase chain reaction (RT-PCR) was used (Xiao, 2005; He, 2014). In seven studies, the TUNEL assay was performed (Wang, 2008; Tang et al., 2010; Mao et al., 2014; Zhang et al., 2015; Liu, 2016; Ko et al., 2018; Tang et al., 2021). In eight studies, immunohistochemistry (IHC) was performed (Wang, 2008; Tang et al., 2010; Mao et al., 2014; Zhang et al., 2015; Liu, 2016; Ko et al., 2018; Liao, 2018; Yu et al., 2018), while in two, an immunofluorescence (IF) assay was conducted (Ko et al., 2018; Tang et al., 2021). Three studies reported brain water content (BWC) (He, 2014; Rao, 2014; Chu et al., 2017). In two studies, correlated factors were determined using an enzyme linked immunosorbent assay (ELISA) (Liao, 2018; Tang et al., 2021). Two studies focused on morphological changes (Rao, 2014; Yu et al., 2018). One study reported results for peripheral blood cells (Tang et al., 2010). One study reported superoxide dismutase (SOD) levels (He, 2014), and another reported brain specific gravity and blood-brain barrier (BBB) permeability (Chu et al., 2017). One study reported the Rotarod test (Ko et al., 2018), and another reported the foot fault test (Tang et al., 2021). The general features of the included studies are listed in Table 1.

**TABLE 1:** Basic characteristics of the included studies.

Author	Type	Species	Anesthetic	Ischemia duration	Time of PF administration	Control group	Experimental group (daily dosage, approach, duration)	Outcome measures	Proposed mechanism
Xiao, (2005)	Doctoral thesis	Rat/M/SD 220–250 g	Chloral hydrate	90 min	48 h before MCAO	NS	40 mg/kg,ip, 24 h	1.CIS,2. NSS,3.RT-PCR(COX-2↓), 4.WB(COX-2↓)	Activation of adenosine A1 receptors and downregulation of COX-2
Wang, (2008)	Master’s thesis	Rat/M/SD 250 ± 30 g	Chloral hydrate	90 min	30 min before MCAO	NS	60 mg/kg,ip, 24 h	1.CIS,2. NSS,3.TUNEL,4.IHC(FAS↓, TNF-α↓), 5.WB(P-P38↓, iNOS↓)	Anti-apoptosis, downregulation of p-p38, iNOS, FAS, and TNF-α
Tang et al. (2010)	Journal	Rat/M/SD 300–350 g	Chloral hydrate	90 min	10 min before MCAO/30 min after MCAO	PBS	20 mg/kg,iv, 24 h	1.NSS, 2. CIS, 3.IHC(ED1↓, IL-1β↓, TNF-α↓, ICAM-1↓, MPO↓),4.TUNEL	Anti-inflammation and anti-apoptosis
He, (2014)	Master’s thesis	Rat/M/SD 250–300 g	Chloral hydrate	90 min	48 h before MCAO	NS	20 mg/kg,ip, 72 h	1.CIS,2. NSS,3.BWC,4.SOD↑,5.RT-PCR(Nrf2↑),6.WB(Nrf2↑)	Anti-oxidative stress, activation of SOD, and upregulation of the Nrf2 pathway
Mao et al. (2014)	Journal	Rat/M/SD 250 ± 10 g	Chloral hydrate	90 min	3d before MCAO	PBS	200 mg/kg,ig, 24 h	1.NSS.2.TUNEL.3.IHC(CHOP↓)	Anti-apoptosis, downregulation of CHOP
Rao, (2014)	Master’s thesis	Rat/M/SD 260–300 g	Chloral hydrate	90 min	30 min after MCAO	NS	40 mg/kg (20 mg/kg,bid), ip, 24 h	1.NSS,2.CIS,3.BWC.4.Morphological changes	Downregulation of arachidonic acid expression via cyclooxygense pathways, activation of CBR2
Zhang et al. (2015)	Journal	Rat/M/SD 280 ± 20 g	Chloral hydrate	2 h	2 h after MCAO	NS	10 mg/kg (5 mg/kg,bid),ip,7d	1.NSS,2.CIS,3.IHC(NeuN↑, GFAP↑, MAP-2↓),4.TUNEL	Deactivation of astrocytes and anti-apoptosis
Liu, (2016)	Journal	Rat/M/SD 250–300 g	Chloral hydrate	24 h	30 min before MCAO	NS	60 mg/kg,ip, 24 h	1.NSS,2.CIS,3.TUNEL,4.IHC (Bcl-2↑, Bax↓)	Anti-apoptosis by downregulation Bax and activation Bcl-2
Chu et al. (2017)	Journal	Rat/M/SD 280–300 g	Chloral hydrate	90 min	1 h after I/R	NM	10 mg/kg (5 mg/kg), bid,ip,7d	1.NSS,2.BWC,3.Brain specific gravity, 4.BBB permeability,5.WB(Cx43↓,AQP4↓,p-JNK↑,p-ERK↔,p-p38↔), 6.IF(AQP4↓)	Downregulation Cx43 and AQP4 via JNK pathway activation
Liao, (2018)	Master’s thesis	Rat/M/SD 200 ± 20 g	Chloral hydrate	1 h	8 h after I/R	NS	5 mg/kg,iv,7d	1.NSS,2.CIS,3.ELISA(IL-1β↓, TNF-α↓), 4.IHC(NF-kB/P65↓)	Downregulation NF-kB pathway, anti-inflammation
Yu et al. (2018)	Journal	Rat/M/SD 180–220 g	Pentobarbital sodium	1 h	6 h after I/R	NS	5 mg/kg,iv,7d	1.NSS,2.CIS,3.Morphological changes, 4.IHC(p-Akt↑)	Activation PI3K/Akt signaling pathway
Ko et al. (2018)	Journal	Rat/M/SD 250–350 g	Isoflurane	15 min	24 h after MCAO	NM	20 mg/kg,ip,6d	1.NSS, 2.Rotarod test, 3.IHC( nAChRs α4β2↓, Ki67↑), 4.IF(CD68↑, nAChR α7↑), 5.TUNEL	Anti-apoptosis and promotion of neurogenesis
Tang et al. (2021)	Journal	Rat/M/SD 200–250 g	Chloral hydrate	2 h	2 h after MCAO	Vehicle (PBS + DMSO)	10 mg/kg,ip,14d	1.NSS,2.foot-fault test,3.WB(Iba-1↓, JNK↔,p-JNK↓,nuclear P65↓),4.ELISA(TNF-α↓, IL-1β↓ and IL-6↓),5.IF(Iba-1↓, vWF↑,DCX↑,P65↓),6.TUNEL	Anti-inflammation and promotion of neurogenesis

AKT, Protein kinase B; AQP4, Aquaporin4; BAX, BCL-2, associated X; BBB, Blood-brain barrier; BCL-2, B-cell lymphoma-2; Bid, Bis in di; BWC, brain water content; CBR2, Cannabinoid 2 receptors; CHOP, C/EBP, homologous protein; CIS, cerebral infarction size; COX-2, Cyclooxygenase 2; Cx43, Connexin43; d, Day; DCX, doublecortin; ED1, Mouse anti rat CD68; ELISA, Enzyme linked immunosorbent assay; ERK, Extracellular signal-regulated kinase; FAS, fas cell surface death receptor; GFAP, glial fibrillary acidic protein; h, Hour; i.g, Irrigation; i.p., intraperitoneal; i.v., intravenous; Iba-1, Ionized calcium-binding adapter molecule 1; ICAM-1, Intercellular adhesion molecule-1; IF, immunofluorescence; IHC, immunohistochemistry; IL-1β, Interleukin-1β; IL-6, Interleukin-6; iNOS, inducible nitric oxide synthase; JNK, c-Jun N-terminal kinase; Ki67, Mitotic cell marker; MAP-2, Microtubule-associated protein 2; MCAO, middle cerebral artery occlusion; min, Minute; MPO, myeloperoxidase; nAChRsα4β2, α4β2 nicotinic acetylcholine receptors; nAChRα7, α7 nicotinic acetylcholine receptor; NeuN, Neuron-specific nuclear; NF-κB/P65, Nuclear transcription factor-kappa B; NM, not mentioned; Nrf2, Nuclear factor erythroid 2-related factor 2; NS, normal saline; NSS, neurological severity score; p-AKT, Phosphorylated AKT; PBS, Phosphate-buffered saline; p-ERK, Phosphorylated ERK; PI3K, Phosphoinositide 3-kinases; p-JNK, Phosphorylated JNK; p-P38, Phosphorylated P38; RT-PCR, Reverse transcription-polymerase chain reaction; SD, Sprague-Dawley; SOD, Superoxide dismutase; TNF-α, Tumor necrosis factor-α; vWF, von willebrand factor; WB, Western blot. ↑, upregulated; ↓, downregulated; ↔, No difference.

### Methodological Quality of the Included Studies

**FIGURE 3:**
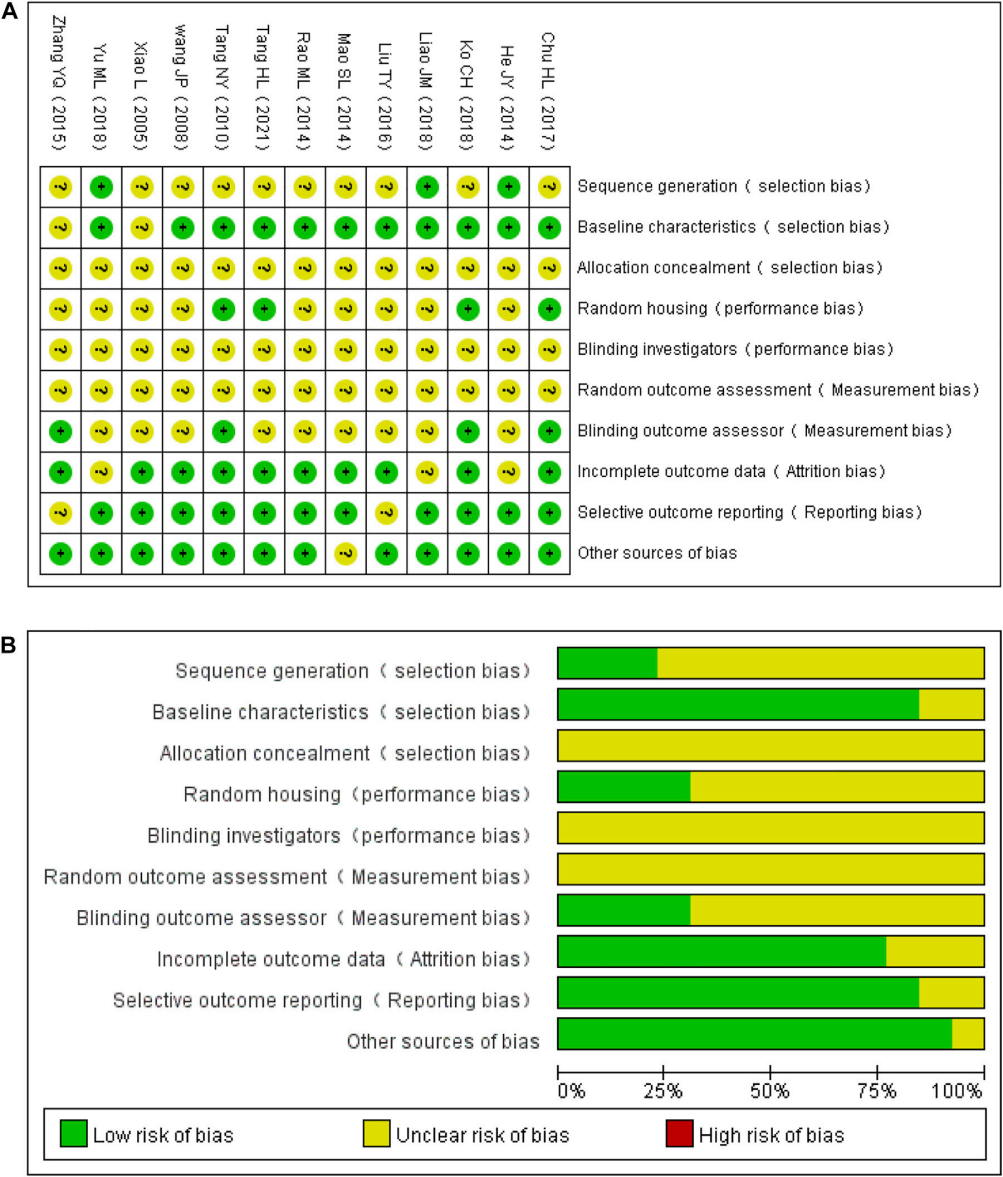
Evaluation of the literature quality results obtained through SYRCLE’s risk of bias based on the Cochrane tool. **(A)** Risk of bias summary. **(B)** Risk of bias graph.

The quality scores of studies ranged from 3 to 6. Two studies did not report random grouping (Tang et al., 2010; Tang et al., 2021). Only three studies out of 11 reported the exact randomization method (He, 2014; Liao, 2018; Yu et al., 2018), despite the fact that 11 studies reported randomization (Xiao, 2005; Wang, 2008; He, 2014; Mao et al., 2014; Rao, 2014; Zhang et al., 2015; Liu, 2016; Chu et al., 2017; Ko et al., 2018; Liao, 2018; Yu et al., 2018). In five studies (Tang et al., 2010; Rao, 2014; Chu et al., 2017; Ko et al., 2018; Tang et al., 2021), the modeling method was assessed using doppler analysis, and in six studies (Wang, 2008; He, 2014; Mao et al., 2014; Liu, 2016; Liao, 2018; Yu et al., 2018), NSS was used to guarantee the unification of experimental baseline standards. In the remaining studies, the modeling method was not assessed. Four studies (Tang et al., 2010; Chu et al., 2017; Ko et al., 2018; Tang et al., 2021) reported the feeding environment of the animals. However, none of the studies reported allocation concealment, blinding of investigators, or random outcome assessments. In four studies (Tang et al., 2010; Zhang et al., 2015; Chu et al., 2017; Ko et al., 2018), assessors were blinded to outcomes. The data reported in three studies were incomplete (He, 2014; Liao, 2018; Yu et al., 2018). Results were inconsistent with the research methods in two studies (Zhang et al., 2015; Liu, 2016). One study reported the supply of new animals (Mao et al., 2014). The general features of the included studies are shown in Table 2 and Figure 3.

**TABLE 2:** The research quality of the included studies.

Study	①	②	③	④	⑤	⑥	⑦	⑧	⑨	⑩	Scores
Xiao, (2005)	0	0	0	0	0	0	0	1	1	1	3
Wang, (2008)	0	1	0	0	0	0	0	1	1	1	4
Tang et al. (2010)	0	1	0	1	0	0	1	1	1	1	6
He, (2014)	1	1	0	0	0	0	0	0	1	1	4
Mao et al. (2014)	0	1	0	0	0	0	0	1	1	0	3
Rao, (2014)	0	1	0	0	0	0	0	1	1	1	4
Zhang et al. (2015)	0	0	0	0	0	0	1	1	0	1	3
Liu, (2016)	0	1	0	0	0	0	0	1	0	1	3
Chu et al. (2017)	0	1	0	1	0	0	1	1	1	1	6
Liao, (2018)	1	1	0	0	0	0	0	0	1	1	4
Yu et al. (2018)	1	1	0	0	0	0	0	0	1	1	4
Ko et al. (2018)	0	1	0	1	0	0	1	1	1	1	6
Tang et al. (2021)	0	1	0	1	0	0	0	1	1	1	5

①Sequence generation; ②Baseline characteristics; ③Allocation concealment; ④Random housing; ⑤Blinding of investigators; ⑥Random outcome assessment; ⑦Blinding of assessors to the outcomes; ⑧Incomplete outcome data; ⑨Selective outcome reporting; ⑩Other sources of bias.

### NSS

**FIGURE 4:**
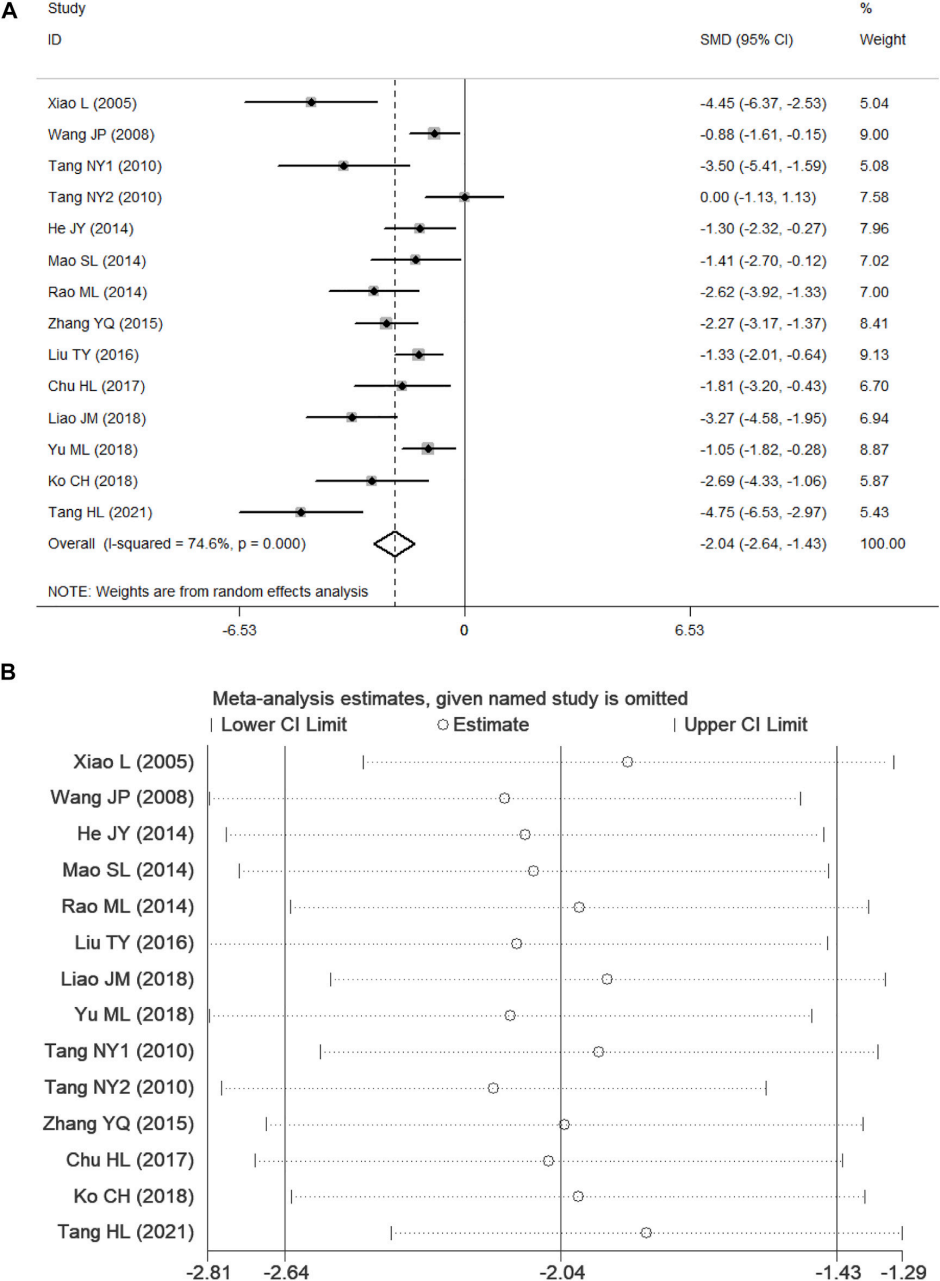
Forest plots of PF for NSS. **(A)** Effects of PF on decreasing the NSS in comparison with the control group; **(B)** sensitivity analysis of PF for NSS.

According to *p* < 0.1 and I^2^ > 50%,an analysis of NSS data in 13 studies (Xiao, 2005; Wang, 2008; Tang et al., 2010; He, 2014; Mao et al., 2014; Rao, 2014; Zhang et al., 2015; Liu, 2016; Chu et al., 2017; Ko et al., 2018; Liao, 2018; Yu et al., 2018; Tang et al., 2021) showed significant heterogeneity among the results of the studies (*p* = 0.000, I^2^ = 74.6%). A random-effects model was used for the analyses, and in comparison with the control group, PF was shown to reduce the NSS (SMD = −2.04, 95% CI = [−2.64, −1.43], *p* = 0.000). After sensitivity analysis of the included studies, PF was still shown to reduce the NSS in comparison with the control group (Figure 4). Subgroup analysis indicated that the improvement in the NSS summarized estimated value did not depend on the PF intervention time, duration, daily dosage, and ischemia time (Table 3). Meta-regression did not demonstrate a prominent influence of the covariates (intervention time, duration, daily dosage, ischemia time, sample size, route of administration and anesthetic) on the effects of PF (Table 4).

**TABLE 3:** Study characteristics accounting for heterogeneity in the NSS subgroup analysis.

Analysis	References	Fixed-effects model, HR (95% CI)	*p*	Random-effects model HR (95% CI)	*p*	I^2^ (%)	ph
NSS	Chu et al. (2017), He, (2014), Ko et al. (2018), Liao, (2018), Liu, (2016), Mao et al. (2014), Rao, (2014), Tang et al. (2021), Tang et al. (2010), Wang, (2008), Xiao, (2005), Yu et al. (2018), Zhang et al. (2015)	−1.657(−1.943, −1.370)	0.000	−2.036 (−2.638, −1.435)	0.000	74.6	0.000
Subgroup 1							
pre-treatment	He, (2014), Liu, (2016), Mao et al. (2014), Tang et al. (2010), Wang, (2008), Xiao, (2005)	−1.430(−1.835–1.025)	0.000	−1.806 (−2.620, −0.992)	0.000	69.5	0.006
post-treatment	Chu et al. (2017), Ko et al. (2018), Liao, (2018), Rao, (2014), Tang et al. (2021), Tang et al. (2010), Yu et al. (2018), Zhang et al. (2015)	−1.883 (−2.288, −1.479)	0.000	−2.198 (−3.107, −1.289)	0.000	78.3	0.000
Subgroup 2							
Duration = 24 h	Liu, (2016), Mao et al. (2014), Rao, (2014), Tang et al. (2010), Wang, (2008), Xiao, (2005)	−1.387 (−1.778, −0.995)	0.000	−1.791 (−2.677, −0.906)	0.000	76.6	0.002
Duration >24 h	Chu et al. (2017), He, (2014), Ko et al. (2018), Liao, (2018), Tang et al. (2021), Yu et al. (2018), Zhang et al. (2015)	−1.967(−2.387, −1.547)	0.000	−2.291 (−3.131, −1.452)	0.001	72.1	0.001
Subgroup 3							
Daily dosage ≤10 mg/kg	Liao, (2018), Tang et al. (2021), Yu et al. (2018)	−1.994 (−2.614, −1.373)	0.000	−2.912 (−5.104, −0.721)	0.000	89.3	0.000
Daily dosage ≤20 mg/kg	Chu et al. (2017), He, (2014), Ko et al. (2018), Tang et al. (2010), Zhang et al. (2015)	−1.681 (−2.173, −1.189)	0.000	-1.802 (-2.712,-0.892)	0.000	68.1	0.008
Daily dosage >20 mg/kg	Liu, (2016), Mao et al. (2014), Rao, (2014), Wang, (2008), Xiao, (2005)	−1.478(−1.906, −1.051)	0.000	−1.873 (−2.790, −0.957)	0.000	73.4	0.005
Subgroup 4							
Ischemia time ≤90 min	Chu et al. (2017), He, (2014), Ko et al. (2018), Liao, (2018), Mao et al. (2014), Rao, (2014), Tang et al. (2010), Wang, (2008), Xiao, (2005), Yu et al. (2018)	−1.536 (−1.878,-1.193)	0.000	−1.905 (−2.595, −1.216)	0.000	72.5	0.000
Ischemia time >90 min	Liu, (2016), Tang et al. (2021), Zhang et al. (2015)	−1.938(-2.461, −1.416)	0.000	−2.562 (−4.076, −1.048)	0.001	84.8	0.001

**TABLE 4:** Meta-regression analysis of potential sources of heterogeneity.

Heterogeneity factor	Coefficient	SE	t	p-value	95% CI
Intervention time	1.255402	2.6493	0.47	0.660	−6.100234, 8.611038
Duration	2.497039	4.00392	0.62	0.567	−8.619626, 13.6137
Daily dosage	2.375791	3.072568	0.77	0.483	−6.155026, 10.90661
Ischemia time	−0.0238451	2.307099	−0.01	0.992	−6.429379, 6.381689
Sample size	1.095033	1.722891	0.64	0.560	−3.688479, 5.878546
Route of administration	0.6597497	1.856658	0.36	0.740	−4.495158, 5.814658
Anesthetic	0.5705356	2.355482	0.24	0.821	−5.96933, 7.110401

### CIS

**FIGURE 5:**
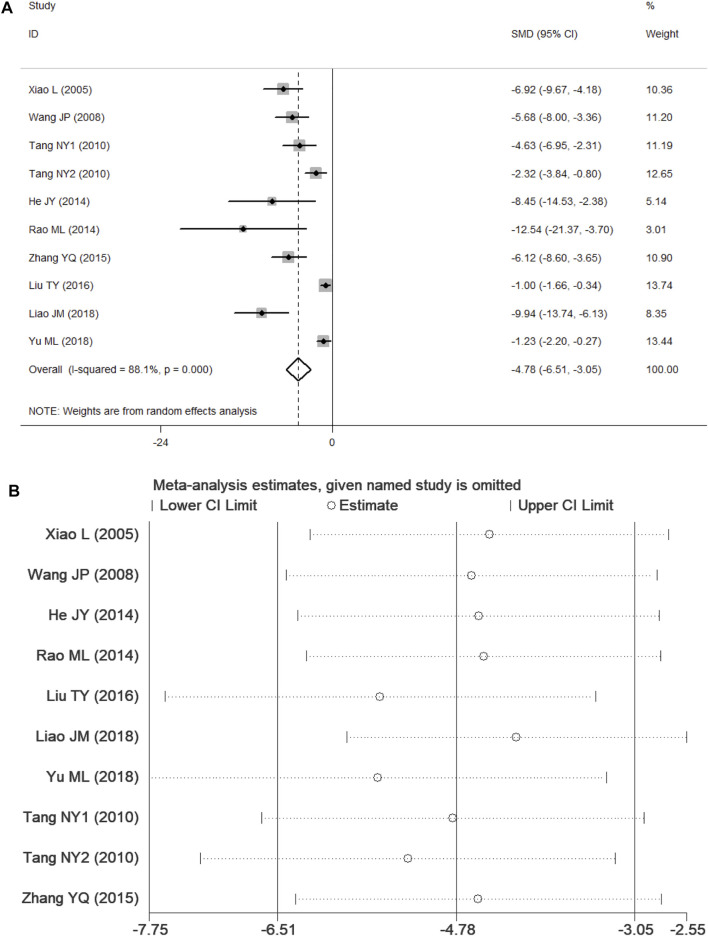
Forest plots of PF for CIS. **(A)** Effects of PF on decreasing the CIS in comparison with the control group; **(B)** sensitivity analysis of PF for CIS.

Nine studies (Xiao, 2005; Wang, 2008; Tang et al., 2010; He, 2014; Rao, 2014; Zhang et al., 2015; Liu, 2016; Liao, 2018; Yu et al., 2018) presented CIS data. According to *p* < 0.1 and I^2^ > 50%, and the results showed significant heterogeneity (*p* = 0.000, I^2^ = 88.1%). In comparison with the control group, PF was shown to reduce the CIS in the random-effects model (SMD = −4.78, 95% CI = [−6.51, −3.05], *p* = 0.000). After sensitivity analysis of the included studies, PF was still shown to reduce the CIS in comparison with the control group (Figure 5).

### BWC

**FIGURE 6:**
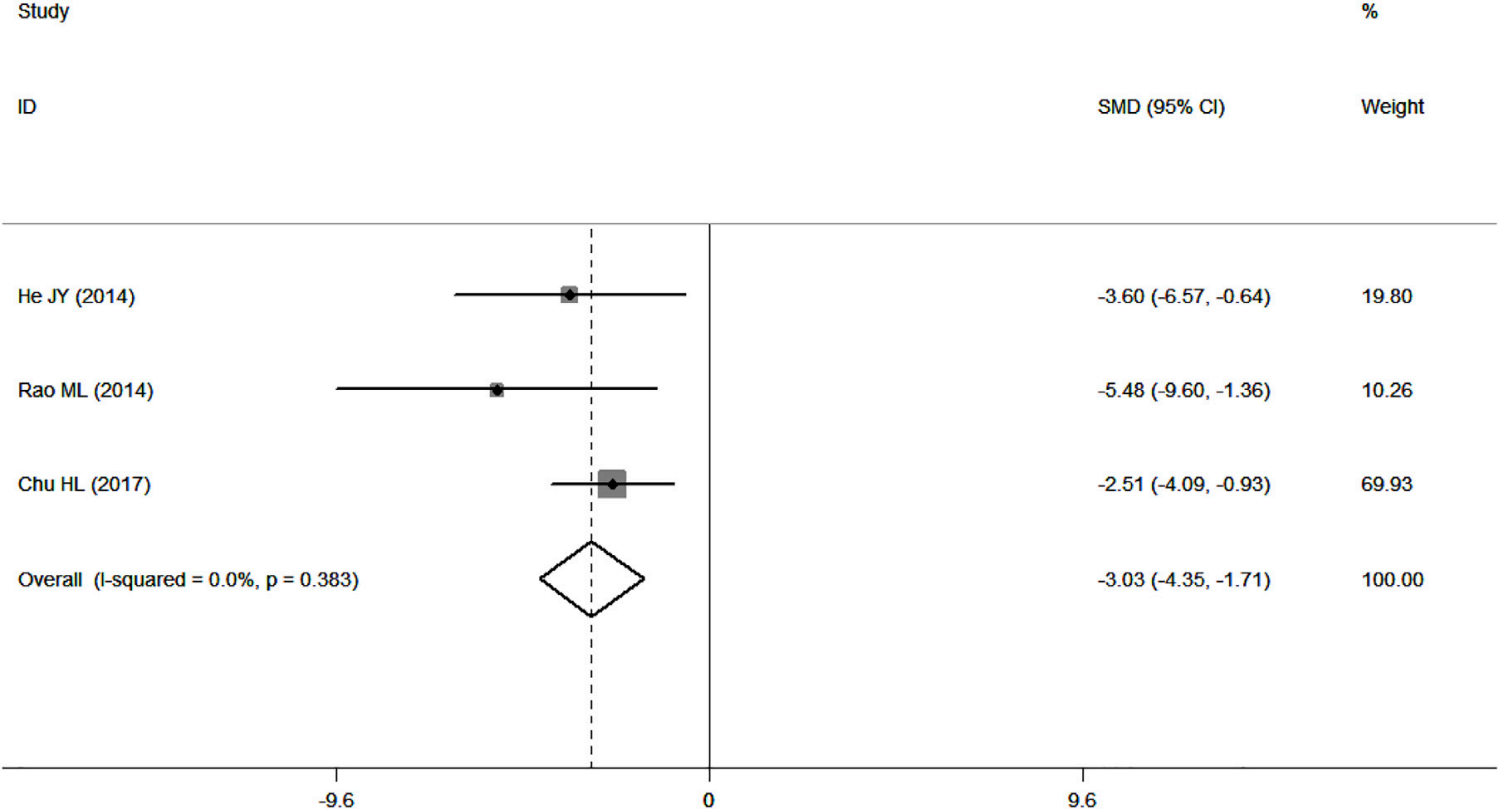
Forest plots of PF for BWC.

Three studies (He, 2014; Rao, 2014; Chu et al., 2017) presented data for BWC, and the results showed no heterogeneity according to *p* > 0.1 and I^2^ ≤ 50% (*p* = 0.383, I^2^ = 0.0%). In comparison with the control group, PF was shown to alleviate BWC in analyses with the fixed-effects model (SMD = −3.03, 95% CI = [−4.35, −1.71], *p* = 0.000; Figure 6).

### Other Outcomes

**FIGURE 7:**
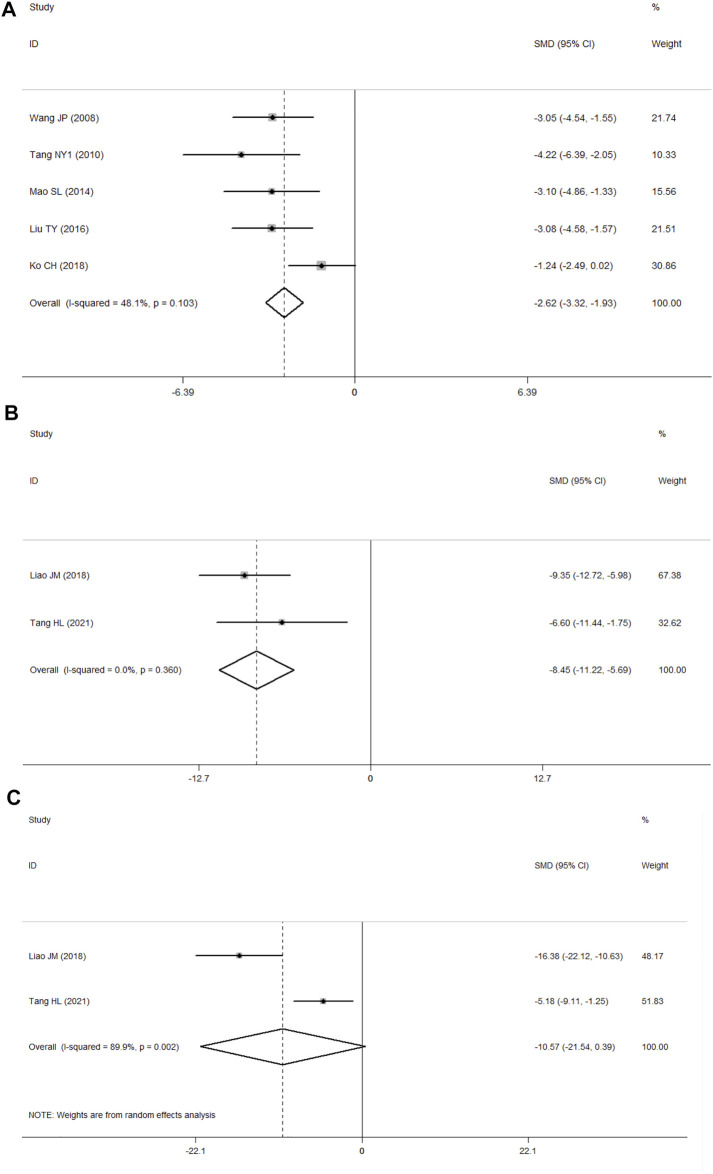
**(A)** Forest plots of PF for TUNEL staining; **(B)** forest plots of PF for IL-1β; **(C)** forest plots of PF for TNF-α.

Seven studies (Wang, 2008; Tang et al., 2010; Mao et al., 2014; Zhang et al., 2015; Liu, 2016; Ko et al., 2018; Tang et al., 2021) presented the results of TUNEL staining. Among these, one study (Zhang et al., 2015) was ruled out because the data were not available, and another study (Tang et al., 2021) was ruled out because of substantial heterogeneity in the data. A fixed-effects model was used with the last five studies (Wang, 2008; Tang et al., 2010; Mao et al., 2014; Liu, 2016; Ko et al., 2018) because of no heterogeneity among them according to *p* > 0.1 and I^2^ ≤ 50% (*p* = 0.103, I^2^ = 48.1%). In comparison with the control group, PF was shown to inhibit apoptosis (SMD = −2.62, 95% CI = [−3.32, −1.93], *p* = 0.000; Figure 7A).

Two studies presented the results of ELISA (Liao, 2018; Tang et al., 2021), of which one reported the findings for interleukin-1β (IL-1β), tumor necrosis factor-α(TNF-α), and interleukin-6(IL-6) (Tang et al., 2021), while the other one reported data for IL-1β and TNF-α (Liao, 2018). According to *p* > 0.1 and I^2^ ≤ 50%, the IL-1β results showed no heterogeneity (*p* = 0.360, I^2^ = 0.0%). In comparison with the control group using the fixed-effects model, PF was shown to decrease the level of IL-1β (SMD = −8.45, 95% CI = [−11.22, −5.69], *p* = 0.000; Figure 7B), and the TNF-α results showed significant heterogeneity (*p* = 0.002, I^2^ = 89.9%) according to *p* < 0.1 and I^2^ > 50%. In analyses with a random-effects model, in comparison with the control group, PF was shown to decrease the level of TNF-α, but the difference was not statistically significant (SMD = −10.57, 95% CI = [−21.54, 0.39], *p* = 0.059; Figure 7C).

Two studies reported morphological changes (Rao, 2014; Yu et al., 2018). In comparison with the control group, most nerve cells in the hippocampus of cornu ammonis 1(CA1) in the PF group were characterized by structural integrity, light morphological changes, and less karyopyknosis. In other analyses, one study reported that PF could improve the activity of SOD in the MCAO model (He, 2014); one study reported that PF could increase brain-specific gravity and reduce BBB permeability in the MCAO model (Chu et al., 2017); one study (Ko et al., 2018) reported the findings for the Rotarod test and one study (Tang et al., 2021) reported the findings for the foot-fault test, and the results of both tests showed that PF could improve neurological symptoms. The results of WB (Xiao, 2005; Wang, 2008; He, 2014; Chu et al., 2017; Tang et al., 2021), RT-PCR (Xiao, 2005; He, 2014), IHC (Wang, 2008; Tang et al., 2010; Mao et al., 2014; Zhang et al., 2015; Liu, 2016; Ko et al., 2018; Liao, 2018; Yu et al., 2018), and IF (Ko et al., 2018; Tang et al., 2021) are shown in Table 1. Complete data can be found in the Supplementary Table S1 and the PRISMA 2020 Checklist is in the Supplementary Table S2.

### Publication Bias

**FIGURE 8:**
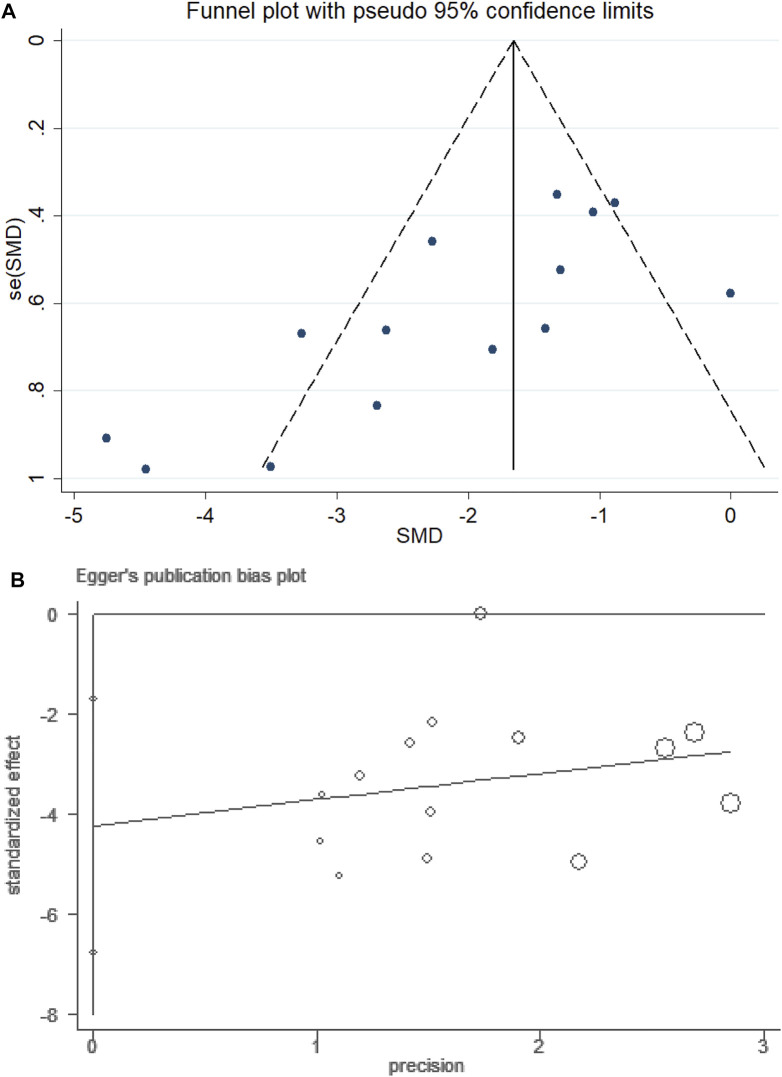
Funnel plot of PF for NSS. **(A)** Assessment of publication bias in a funnel plot. **(B)** Bias assessment plot by Egger’s test.

For the NSS subset, Egger’s linear regression test was performed, and it indicated the possibility of publication bias (P > ItI = 0.004 for Egger’s test). The adjusted random-effects pooled HR of −2.036 (95% CI, −2.638 to −1.435), obtained using the trim-and-fill method, was unchanged because no trimming was performed (Figure 8).

## Discussion

The main targets in the treatment of acute stroke are recovery of cerebral blood flow, and mechanical thrombectomy and intravenous thrombolysis are the main therapeutic strategies at present. However, the narrow time windows and contraindications are major obstacles to the universal application of these therapeutic approaches. On the other hand, vascular recanalization and I/R are often interrelated (Smith et al., 2019). The supply of oxygen and glucose is reduced after the onset of cerebral ischemia, and the recovered oxygen-rich blood from the ischemic damaged brain tissue would offer the necessary substrate for the generation of reactive oxygen species (ROS) if recanalization occurs after the key time window (Eltzschig and Eckle, 2011). ROS can not only lead to direct cell injuries and apoptosis but can also trigger the activation of adaptive immunity and brain innate immunity. This process can induce the formation of various destructive immunological mediators and effectors, eventually creating a vicious circle (Mizuma and Yenari, 2017). The mechanisms underlying ischemic stroke have been explored in depth over many years, although clinical studies did not often yield good outcomes. Thus, there is a constant need for the identification of novel neuroprotective agents (Chamorro, 2018). The neuroprotective effect of PF may be relevant to some molecular mechanisms, such as the mode of cell death, inflammation, oxidative stress and epigenetics.

Cell death triggered by I/R injury not only consists of cell necrosis but also includes programmed cell deaths such as apoptosis (Liao et al., 2020), autophagia (Shen et al., 2021), and pyroptosis (Gou et al., 2021). These procedures are monitored by multiple signaling mechanisms by interfering with a relevant signal pathway to save damaged cells (Datta et al., 2020). Apoptotic pathways consist of the intrinsic apoptotic pathway mediated by mitochondria and the extrinsic apoptotic pathway mediated by death receptors, among which caspase and the B-cell lymphoma 2 (BCL-2) protein family are major molecules (Arya and White, 2015). Studies have shown that PF can maintain the integrity of the mitochondrial membrane, reduce the level of BCL-2 associated X (BAX), BCL-2 associated agonist of cell death (BAD), downstream caspase-3 and caspase-9, and increase the levels of BCL-2 and B cell lymphoma-extra large (BCL-XL), thereby showing anti-apoptotic effects (Chen et al., 2017; Cong et al., 2019; Liu et al., 2021; Zhang and Yang, 2021). In autophagia, which is under the control of autophagia-related genes, lysosomes are used to degrade unnecessary or damaged organelles and proteins to maintain cellular homeostasis. The activation conditions of I/R injuries (such as energy deprivation, oxidative stress and endoplasmic reticulum stress) could result in autophagia (Wu et al., 2018). Appropriate autophagia could offer nerve protection and facilitate improvements in clinical results by significantly decreasing the levels of neurons, glial, and endothelial cells (Ajoolabady et al., 2021). PF has been shown to promote autophagy by regulating the lipidation of microtubule associated protein 1 light chain 3 (LC3-II) (Cao et al., 2010). Pyroptosis is a kind of programmed death of inflammatory cells, which could cause lysis and oligomerization of gasdermin protein family members, including gasdermin D (GSDMD), cell perforation, or even worse, cell death. The process is triggered by the activation of inflammasome-mediated caspases, including caspase-1 (Tuo et al., 2021). In comparison with apoptosis, pyroptosis occurs more rapidly and is associated with a greater release of proinflammatory factors (Tsuchiya, 2021). PF has been shown to alleviate astrocyte pyroptosis caused by hypoxia through the Caspase 1/GSDMD signal pathway (She et al., 2019).

Cerebral I/R injury triggers inflammation without microorganism participation, although the inflammation shows features common with those caused by invading pathogens. This immunologic response involves the collection and activation of pattern recognition receptors, including Toll-Like receptors (TLRs), immune cells of the innate and adaptive immune systems, and the activation of complement systems to pass signal events. Because these responses may have adverse consequences, targeted immune activation has become an emerging treatment modality for I/R injuries (Carbone et al., 2019; Stoll and Nieswandt, 2019). Some studies have shown that PF may have anti-inflammatory effects through the signal pathway of TLR4- Myeloid differentiation factor 88 (MyD88)/Nuclear transcription factor-kappa B(NF-κB) (Zhang et al., 2017; Yang et al., 2021) and Janus kinase 2 (JAK2)/Signal transducer and activator of transcription 3(STAT3) (Zhang and Yang, 2021).

Oxidative stress, which is generated as a result of elevated levels of ROS and reactive nitrogen species and reduced levels of antioxidants, can cause damage to cell components, including proteins, lipids, and DNA (Zhao et al., 2016). Malondialdehyde (MDA), as the end product of lipid oxidation, can induce crosslinking polymerization of proteins, nucleic acids, and other macromolecules. In addition, due to MDA’s cytotoxicity, the stronger its activity becomes, the stronger the lipid peroxidization, which can trigger oxidative stress damage (Menon et al., 2020). SOD, an important active ingredient in organisms, can eliminate harmful substances and maintain good metabolic conditions. The lower the levels of SOD, the weaker the cells’ ability to prevent oxidative damage (Cherubini et al., 2000). Glutathione (GSH), a tripeptide consisting of γ-amido bonds and sulfydryl, can perform integrated detoxification and antioxidation functions. GSH measurements are also a common index to evaluate antioxidation ability (Higashi et al., 2021). Unsaturated double bonds in cytomembrane phospholipids are easily attacked by oxygen radicals, resulting in the invagination of phosphatidylserine on cytomembranes, incompleteness of cytomembranes, and release of lactate dehydrogenase (LDH) (Bhowmick and Drew, 2017). Studies have shown that PF can improve these targets and alleviate the brain damage (Liu and Wang, 2013; Wang et al., 2020; Wu et al., 2020; Zhang and Yang, 2021).

Epigenetics, the transitive variation of phenotypic characters, is unrelated to DNA changes, but may be influenced by external and environmental factors. These factors can turn on and off genes and thus affect the ways in which cells read genes. There are three primary epigenetic mechanisms: DNA methylation, histone modification, and non-coding RNA (Patsouras and Vlachoyiannopoulos, 2019). (1) Members of the histone deacetylase (HDAC) family compete with histone acetyltransferase (HAT) for the right to control lysine residue acetylation that forms histone, thereby ensuring post-translational acetylation of chromatin and many other non-histones (He et al., 2013). Many studies have reported the neuroprotective roles of HDAC inhibitors in ischemic stroke (Patnala et al., 2017; Brookes et al., 2018), and PF has been shown to reduce ischemic brain injuries triggered by caspase 3-induced HDAC4 nuclear accumulation during stroke (Liu et al., 2021). (2) The common non-coding RNA consists of lncRNAs and miRNAs, and miRNAs have especially attracted considerable attention in cerebral I/R injuries studies in recent years (Ghafouri-Fard et al., 2020). miRNAs can not only influence gene expression by inhibiting mRNA translation or inducing degradation of mRNA, but also act as damage-associated molecular patterns and cofactors activating inflammatory cascades and thrombosis (Hu et al., 2015; Forouzanfar et al., 2019; Mahjoubin-Tehran et al., 2021). Studies show that PF could alleviate brain damage by manipulating miR-210 (Jiang et al., 2021) and miR-135a (Zhai et al., 2019).

This is the first preclinical meta-analysis to investigate the efficacy of PF for cerebral I/R Injury. The findings confirmed that, in comparison with the control group, PF showed improvements in the NSS, CIS, and BWC by modulating a wide range of biological mechanisms such as neuroinflammation, oxidative stress, and apoptosis. The results of subgroup analysis showed that the longer the ischemia duration, the more severe the injury and the better the treatment effect of PF. The effect of PF administered post-MCAO was better than that administered pre-MCAO, but this phenomenon could be explained by the long observation period. Daily dosage ≤10 mg/kg or >20 mg/kg for PF were better than daily dosages ≤20 mg/kg, indicating a “U-Shaped Dose-Response Curve” between PF dosage and therapeutic effect. There are barriers to turning experimental findings into clinically viable therapies, particularly in the research of cerebrovascular disorders. It is important to confirm PF’s efficacy in larger animal models, to evaluate the therapeutic benefit of combination application with other neuroprotective treatments, and to clarify its potential side effects and safety in order to advance PF into clinical trials as soon as feasible.

## Limitations

First, the studies evaluated in this meta-analysis had problems related to nonstandard methodologies and incomplete reports, which may have influenced the effectiveness of our conclusions. None of the included studies mentioned power calculation. The lack of a formal sample size calculation leads to uncertainty about the validity of statistical analysis. Particularly for allocation concealment, blinding methods to address performance bias and random outcome evaluation were not mentioned in any studies. A few studies have also been reported on “random housing.” This could be an issue as cage size, material, placement, bedding, and the number of animals placed in the cage may affect thermoregulation and stress level. Lack of information on these elements could potentially contribute to bias. Second, ischemic stroke shows high complexity and heterogeneity. Stroke experiment models can only cover specific features of multiple diseases (Sommer, 2017). Clinical conditions are more complex; for example, many factors may affect prognosis, including hypertension, diabetes, and atrial fibrillation (Boehme et al., 2017). The design differences between experimental studies and clinical studies can result in a gradual decrease in effectiveness from early clinical trials to phase III trials (Schmidt-Pogoda et al., 2020). Thus, to connect preclinical and clinical studies, the quality of animal research methods requires improvement through more systematic methods for the analysis of experimental data and greater collaboration between clinical and animal researchers. Third, the funnel plot shows high asymmetry, indicating a publication bias in this study. The results of the Egger test further validated this finding. However, the findings using the trim-and-fill method were unchanged because no trimming was performed. However, similar to other meta-analyses, these conclusions are influenced by the fact that preclinical studies are usually published if the analyses with experimental animals yield positive results. The resultant lack of studies showing lack of effectiveness or negative findings can result in overestimation of the overall curative effects.

## Conclusion

This preclinical meta-analysis suggests that PF could alleviate cerebral I/R injuries and potentially serve as a neuroprotective agent. Despite the lack of clinical trial data and potential publication biases, these conclusion are worth consideration.

## Data Availability

The original contributions presented in the study are included in the article/Supplementary Material, further inquiries can be directed to the corresponding author.

## Glossary

AKT: Protein kinase B
AQP4: Aquaporin4
BAD: BCL-2 associated agonist of cell death
BAX: BCL-2 associated X
BBB: Blood-brain barrier
BCL-2: B-cell lymphoma-2
BCL-XL: B cell lymphoma-extra large
Bid: Bis in die
BWC: Brain water content
CA1: Cornu ammonis 1
CBR2: Cannabinoid 2 receptors
CHOP: C/EBP homologous protein
CIS: Cerebral infarction size
COX-2: Cyclooxygenase 2
Cx43: Connexin43
d: Day
DCX: Doublecortin
ED1: Mouse anti rat CD68
ELISA: Enzyme linked immunosorbent assay
ERK: Extracellular signal-regulated kinase
FAS: Fas cell surface death receptor
GFAP: Glial fibrillary acidic protein
GSDMD: Gasdermin D
GSH: Glutathione
h: Hour
HAT: Histone acetyltransferase
HDAC: Histone deacetylase
i.g: Irrigation
i.p.: Intraperitoneal
i.v.: Intravenous
I/R: Ischemia/reperfusion
Iba-1: Ionized calcium-binding adapter molecule 1
ICAM-1: Intercellular adhesion molecule-1
IF: Immunofluorescence
IHC: Immunohistochemistry
IL-1β: Interleukin-1β
IL-6: Interleukin-6
iNOS: Inducible Nitric Oxide Synthase
JAK2: Janus kinase 2
JNK: c-Jun N-terminal kinase
Ki67: Mitotic cell marker
LC3-II: Lipidation of microtubule associated protein 1 light chain 3
LDH: Lactate dehydrogenase
MAP-2: Microtubule-associated protein 2
MCAO: Middle cerebral artery occlusion
MDA: Malondialdehyde
min: minute
MPO: Myeloperoxidase
MyD88: Myeloid differentiation factor 88
nAChRsα4β2: α4β2 nicotinic acetylcholine receptors
nAChRα7: α7 nicotinic acetylcholine receptor
NeuN: Neuron-specific nuclear
NF-κB/P65: Nuclear transcription factor-kappa B
NM: Not mentioned
Nrf2: Nuclear factor erythroid 2-related factor 2
NS: Normal saline
NSS: Neurological severity score
p-AKT: Phosphorylated AKT
PBS: Phosphate-buffered saline
p-ERK: Phosphorylated ERK
PF: Paeoniflorin
PI3K: Phosphoinositide 3-kinases
p-JNK: Phosphorylated JNK
p-P38: Phosphorylated P38
PRISMA: Preferred Reporting Items for Systematic Reviews and Meta-Analyses
ROS: Reactive oxygen species
RT-PCR: Reverse transcription-polymerase chain reaction
SD: Sprague-Dawley
SOD: Superoxide dismutase
STAT3: Signal transducer and activator of transcription 3
TCM: Traditional Chinese medicine
TLRs: Toll-Like receptors
TNF-α: Tumor necrosis factor-α
vWF: Von Willebrand Factor
WB: Western blot
